# A brief, standardized tool for measuring HIV-related stigma among health facility staff: results of field testing in China, Dominica, Egypt, Kenya, Puerto Rico and St. Christopher & Nevis

**DOI:** 10.7448/IAS.16.3.18718

**Published:** 2013-11-13

**Authors:** Laura Nyblade, Aparna Jain, Manal Benkirane, Li Li, Anna-Leena Lohiniva, Roger McLean, Janet M Turan, Nelson Varas-Díaz, Francheska Cintrón-Bou, Jihui Guan, Zachary Kwena, Wendell Thomas

**Affiliations:** 1 Health Policy Project and RTI International, Washington, DC, USA; 2 Health Policy Project, Futures Group,Washington, DC, USA; 3 Global Disease Detection and Response Programme at the U.S. Naval Medical Research Unit No. 3, Cairo, Egypt; 4 Department of Psychiatry and Biobehavioural Sciences, School of Public Health, Semel Institute – Center for Community Health, University of California, Los Angeles, CA, USA; 5 Department of Epidemiology, School of Public Health, Semel Institute – Center for Community Health, University of California, Los Angeles, CA, USA; 6 Faculty of Social Sciences, Centre for Health Economics, University of the West Indies, Port-of-Spain, Trinidad and Tobago; 7 Department of Health Organization and Policy, School of Public Health, University of Alabama at Birmingham, Birmingham, AL, USA; 8 Center for Social Research, Social Sciences Faculty, University of Puerto Rico, San Juan, PR, USA; 9 Fujian Provincial Centre for Disease Control and Prevention, Fuzhou, China; 10 Centre for Microbiology Research (CMR), Kenya Medical Research Institute (KEMRI), Kisumu, Kenya; 11 Caribbean Data Management Systems, Port-of-Spain, Trinidad and Tobago

**Keywords:** stigma, discrimination, measurement, stigma-reduction programmes, monitoring, evaluation, health facilities, HIV, AIDS, HIV stigma

## Abstract

**Introduction:**

Within healthcare settings, HIV-related stigma is a recognized barrier to access of HIV prevention and treatment services and yet, few efforts have been made to scale-up stigma reduction programs in service delivery. This is in part due to the lack of a brief, simple, standardized tool for measuring stigma among all levels of health facility staff that works across diverse HIV prevalence, language and healthcare settings. In response, an international consortium led by the Health Policy Project, has developed and field tested a stigma measurement tool for use with health facility staff.

**Methods:**

Experts participated in a content-development workshop to review an item pool of existing measures, identify gaps and prioritize questions. The resulting questionnaire was field tested in six diverse sites (China, Dominica, Egypt, Kenya, Puerto Rico and St. Christopher & Nevis). Respondents included clinical and non-clinical staff. Questionnaires were self- or interviewer-administered. Analysis of item performance across sites examined both psychometric properties and contextual issues.

**Results:**

The key outcome of the process was a substantially reduced questionnaire. Eighteen core questions measure three programmatically actionable drivers of stigma within health facilities (worry about HIV transmission, attitudes towards people living with HIV (PLHIV), and health facility environment, including policies), and enacted stigma. The questionnaire also includes one short scale for attitudes towards PLHIV (5-item scale, *α =* 0.78).

**Conclusions:**

Stigma-reduction programmes in healthcare facilities are urgently needed to improve the quality of care provided, uphold the human right to healthcare, increase access to health services, and maximize investments in HIV prevention and treatment. This brief, standardized tool will facilitate inclusion of stigma measurement in research studies and in routine facility data collection, allowing for the monitoring of stigma within healthcare facilities and evaluation of stigma-reduction programmes. There is potential for wide use of the tool either as a stand-alone survey or integrated within other studies of health facility staff.

## Introduction

HIV-related stigma is a recognized barrier to HIV testing, disclosure of sero-status, linkage to care and adherence to anti-retroviral treatment (ART) [1–6]. While present in all spheres of life, stigma is particularly damaging within health facilities, where people living with or at risk of HIV must seek essential medical care, including ART. Stigma has been well documented within health facilities around the world [7–13], and in the past decade recognition of the importance of providing stigma-free health services has increased, which has led to progress in developing and testing different tools and intervention models for reducing stigma in such settings. These advances, however, have yet to be institutionalized as routine practice or implemented on a large scale.

Scale-up of stigma-reduction programmes in healthcare settings has been slow in part due to the lack of a brief, standardized tool for measuring stigma that works across diverse HIV prevalence, language and healthcare settings. While there exist a few validated research tools [9, 13–17], further use of them in research, evaluation or routine monitoring is hindered by several factors. Most of the tools have been tested in only one country or language, and ease of translation, understandability and local relevance of the tools across diverse contexts is unknown. In addition, though the validated tools often ask similar questions that capture the same stigma domains, the combination of items, the specific question wording and response categories vary. As a result, deciding which tool or items to use can be difficult. In addition, these variations pose challenges for national and/or global reporting systems that seek to track stigma within health facilities in a systematic, comparable way and over time.

Most validated tools focus exclusively on medical staff (e.g., doctors and nurses). However, studies have shown that people living with HIV (PLHIV) also encounter stigma and discrimination from administrators and non-medical staff [10]. Therefore, it is important to address and measure stigma among all levels of facility staff, including non-clinical personnel. Furthermore, most tools were developed for stigma-specific research studies and tend to be long and difficult to incorporate as a module into broader research or evaluation studies or to utilize for routine monitoring purposes.

To fill this measurement gap, a collaborative international effort led by the Health Policy Project (HPP) and composed of a broad range of individuals representing international programme-implementing agencies, university and non-university-based researchers, the global network of PLHIV (GNP + ) and UNAIDS, developed, tested and refined two brief tools for measuring HIV stigma among all levels of health facility staff. The first of these tools, the focus of this article, is tailored to evaluation and research needs. The second is suited for monitoring and situations where there are limited resources to collect data; it is a shorter version of the first [18]. Building on existing measures and with a focus on programmatic action to reduce stigma within health facilities, the tools cover multiple domains that capture enacted (experienced or manifested) stigma as well as the drivers of stigma within health facilities. These drivers include concern about HIV transmission when caring for PLHIV, attitudes towards PLHIV and a supportive health facility environment – a key factor in creating an enabling facility environment that supports staff to offer non-stigmatizing care. An enabling environment includes facility-level policies, safety supplies and training. This article describes a multi-year process and its key result – a brief questionnaire to measure stigma among health facility staff.

## Methods

Our methodological approach included a multi-step process: develop an item pool; review and prioritize items by experts through a workshop to develop the content of the questionnaire; field test the questionnaire in six countries; and analyze the data across sites to examine item performance. The objectives of the analysis across sites were to remove non-performing items and prioritize the remaining items to shorten the questionnaire while ensuring that it still captured the essential domains of stigma within health facilities.

### Item pool

The item pool was developed through a comprehensive literature search using PubMed and other bibliographic databases and included both published and grey literature, as well as some pre-publication questionnaires provided by workshop attendees [8, 9, 13, 16, 17, 19–26]. Seeking as wide an item pool as possible, broad inclusion criteria were applied. Articles, reports or unpublished questionnaires had to include quantitative measures implemented among at least one category of health facility staff and in one of the following domains: fear of HIV infection (including transmission knowledge); attitudes towards PLHIV and key populations (stereotypes and prejudice); observed (enacted stigma) and anticipated discrimination (which includes secondary stigma experienced by health facility staff); and institutional-level facilitators and barriers (facility policy and work environment). No geographic or date restrictions were applied. The final item pool was drawn from 10 peer-reviewed articles, 3 agency reports and 2 unpublished questionnaires. Of these only two were multi-country studies: one was an online study administered only in English and the other was concentrated in East and Southern Africa. In regard to study populations, six questionnaires collected data from a single discipline of medical practitioners, seven from multi-disciplinary medical practitioners and two from all levels of health facility staff. The length of surveys was often difficult to assess comparatively as many published articles only presented final scales, while others presented their full questionnaires. Length ranged significantly from 17 to 81 items or questions, with the majority being on the higher side (40–80 items).

### Content-development workshop

The content-development workshop brought together 22 international stigma measurement and programmatic experts, including PLHIV, in a 2.5-day workshop to review the item pool. This group brought experience from past or current work on stigma-reduction programming or measurement in Brazil, the Caribbean, China, Egypt, India, Kenya, Lesotho, Malawi, Mexico, Puerto Rico, South Africa, Swaziland, Tanzania, Vietnam and Zambia. In small working groups, participants reviewed, assessed and prioritized a comprehensive list of stigma items in key stigma domains that were specified in the item pool. The groups were asked to select items based on seven criteria:

1. Response is clearly attributed to or related to stigma.
2. Applicable across all categories of staff in a facility.
3. Relevant to diverse HIV prevalence, health systems and cultural contexts.
4. Ease of translation.
5. Potential for the questions to be influenced by gender, either of the respondent (healthcare provider) or of the client (if the question asks about actions or attitudes towards a client).
6. Potential of the question to cause/lead/reinforce stigma or discrimination.
7. Overall balance in the set to ensure data on measures are relevant to inform design and measure progress of stigma-reduction programmes.

Based on these criteria, each group was tasked with prioritizing the top two, five and 10 questions in a specific domain, and presented their recommendations back to the larger group for further discussion. Groups were also asked to consider whether there were any gaps in the existing item pool and if so, to propose new questions to fill these. Full workshop deliberations are available in an HPP report [27].

### Measures

Based on the outcomes of the content-development workshop, a questionnaire was developed for field testing [28] that included a background and four core content areas. Table 1 provides all the measures by questionnaire section, including: demographic, job type, and facility-related questions; drivers of stigma; observed and secondary stigma; and measures of stigma towards key populations and pregnant women living with HIV. Enacted stigma in health facilities was also measured by asking respondents whether they had observed specific behaviours or experienced secondary stigma related to caring for patients living with HIV.

**Table 1:** Summary of field-tested measures

Section	Category	Number of questions	Description
Background section	Demographic	6	Age, sex, relationship status, religion, education
	Job duties and facility-related	9	Current position, length of employment in the current job and in healthcare, type and location of facility, type of services provided by respondent, HIV patient case load and types of training received in the past 12 months

Drivers	Health facility policies and work environment	7	Availability of protective supplies (e.g., gloves and post-exposure prophylaxis), training (e.g., on confidentiality), existence and implementation of policies to protect PLHIV, how supportive the facility environment is for staff living with HIV
	Fear	1	Worry of contracting HIV while working with PLHIV; ranging from non-invasive (touching clothing) to invasive (drawing blood). Measures nine different situations (items)
	Attitudes towards PLHIV	1	Attitudes about PLHIV measured through agreement with six different statements (items)
	Shame	2	Two shame questions (e.g., I would be ashamed if I were infected with HIV)
	Willingness to treat key populations	1	Willingness to treat six different key populations including men who have sex with men, sex workers, people who inject drugs. Respondents who indicate unwillingness to treat, then asked whether it was for one of the four reasons

Enacted stigma	Observed	1	Specific behaviours (e.g., denial of care to PLHIV) that have been observed by the respondent in their facility in the last 12 months. Measures eight different behaviours (items)
	Extra infection precautions	1	Extra infection precautions that providers take with PLHIV but not with other patients. Measures six different actions (items)
	Secondary stigma	1	Stigma experienced because of caring for PLHIV (e.g., been avoided by friends or family because of caring for PLHIV); Measures four different actions (items)

Module: stigma towards pregnant women living with HIV among facility staff who care for pregnant women	Fear	1	Worry of contracting HIV during labour and delivery if woman is known to be living with HIV, or if her HIV status is unknown (two items)
	Opinions	1	Attitudes towards pregnant women living with HIV. Measures agreement with seven different attitudinal items.
	Observed	1	Specific behaviours (e.g., neglecting a women living with HIV during labour and delivery) that have been observed in the last 12 months. Measures five different behaviours (items)

### Field testing

The questionnaire was field tested in six sites: China (*n =* 300), Dominica (*n =* 335), Egypt (*n =* 300), Kenya (*n =* 350), Puerto-Rico (*n =* 301) and St. Christopher & Nevis (*n =* 307) between February 2012 and January 2013 (see Table 2 for country-specific dates). Sites for field testing were selected based on groups who participated in the content-development meeting and were able to raise funds to leverage their existing stigma research or programmatic efforts to field test the questionnaire. While the same core questionnaire and minimum sample size (300) were standard across sites, there were variations in types of facilities selected, categories of staff interviewed and methods of survey administration to accommodate site-specific contextual issues (Table 2). A key goal of this process was to develop and test a tool for all levels of facility staff, whether they are clinically trained or not. Therefore, respondents included all staff in a facility, from those who were medically trained at different levels (e.g., doctors, nurses, nurses assistants, dentists, pharmacists) to those who were not (e.g., receptionists, cleaning staff, ward attendants).

**Table 2:** Background information on questionnaire pilot sites

	China	Dominica	Egypt	Kenya	Puerto Rico	St Christopher & Nevis
HIV prevalence	Low	Low	Low	High	Low	Low

Questionnaire language	Chinese	English	Arabic	English, Dholuo, Swahili	Spanish	English

Mode of administration	Self (paper)	Self (paper), interviewer	Interviewer	Self (paper), interviewer	Self (iPad and paper)	Self (paper), interviewer

Date of data collection	April–May 2012	December 2012–January 2013	December 2012	May–June 2012	February–April 2012	November 2012

Ethical approvals from Institutional Review Boards	University of California, Los Angeles (UCLA), the Chinese Center for Disease Control and Prevention (CCDC)	National Human Research Ethics Committee of the Ministry of Health and the Health Media Lab's IRB	Egyptian Ministry of Health, Naval Medical Research Unit No. 3	Kenya Medical Institute (KEMRI) Ethical Review Committee and the University of Alabama at Birmingham (UAB)	University of Puerto Rico's Institutional Committee for the Protection of Human Subjects in Research (CIPSHI)	St. Christopher and Nevis Ministry of Health, Office of the Chief Medical Officer and the Health Media Lab's IRB

Type of facilities	Government County-level Hospitals	National Referral & District Hospitals Health Centers Clinics	Government Infectious Disease Hospital	Government District & Sub-district Hospitals, Health Centers, Dispensaries	Government HIV and STD Clinics, Private Hospitals and Clinics, Religious Community Based Organizations	National Referral & District Hospitals Health Centers Clinics

Number of respondents	300	335	300	350	301	307

Type of respondents^1^	Clinical	Clinical and non-clinical	Clinical and non-clinical	Clinical and non-clinical	Clinical and non-clinical	Clinical and non-clinical

Gender of respondents	Female: 65%; Male: 35%	Female: 82.1%; Male: 17.9%	Female: 74.7%; Male: 25.3%	Female: 56.3%; Male: 43.7%	Female: 72.8%; Male: 27.2%	Female: 81.9%; Male: 18.1%

1 Clinical staff includes those who are medically trained like doctors, nurses, nurse assistants, dentists, pharmacists, and non-clinical staff includes those who were not like receptionists, cleaning staff, ward attendants.

Questionnaires were self- or interviewer-administered, depending on literacy levels, respondent comfort levels with self-completion of the questionnaires, and site-specific contextual needs (Table 2). Interviewers introduced themselves, explained the survey, obtained informed consent and answered any questions that arose in the process of self-completion of the questionnaire. Confidentiality of responses was maintained by not collecting any personal identifiers and by respondents placing completed questionnaires in a sealed envelope or box. Each site obtained ethical clearance from their respective relevant country-level and institutional-level review boards (Table 2).

### Data analysis

Data entry and initial data cleaning were completed at each site and then sent to the global coordinating group for further cleaning and merging into a single, combined data set.

All analyses are conducted in STATA.SE, Version 12 [29]. Performance of the survey items across the six sites was assessed through both examination of psychometric properties and consideration of contextual issues. Initial analysis was conducted by the global coordinating team in preparation for the 2.5-day cross-site analysis workshop that brought together all the principal investigators for each site. During the workshop, the full team considered and discussed several aspects of each question when determining which ones to keep in the brief questionnaire. These aspects included:

1. Variable distributions by country to ascertain reasonable variability in responses.
2. Each site's experience implementing the questions.
3. Exploratory factor analysis or principle component analysis.

These three aspects were reviewed simultaneously and given equal weight when deciding the items that remained in the brief questionnaire.

Exploratory factor analysis was used when exploring the scale associated with attitudes towards PLHIV. For each country, we first ran exploratory factor analysis followed by a scree plot for eigenvalues to determine the number of factors in the scale. We considered potential items for removal if their factor loading was less than 0.35. Scale reliability was analyzed with Cronbach's alpha. Alphas of at least 0.7 are typically used as a cutoff to establish internally consistent scales. Given the goal to reduce the number of items in the scales and to make comparisons among groups, it was resolved to go with a lower yet acceptable cutoff of 0.6 at each of the sites [30–32] for the attitudinal scale.

The worry of HIV infection items included a “not applicable” response category because the items were related to job duty. If a respondent did not typically conduct the activity, they were prompted to select “not applicable.” As a result, when we ran exploratory factor analysis and scree plots by country on the nine items, our sample sizes were reduced considerably; in Egypt we found that none of the respondents answered all items. Therefore, we did not use factor analysis as a method for reducing items, but instead identified two criteria: all staff type can at least answer one item and identify a range of items based on procedure invasiveness to capture/reflect a continuum of worry.

Principle component analysis was used to reduce items in the remaining sections: observed stigma, secondary stigma, and health facility policies and work environment.

Combined with the above analyses, each site's experience implementing the questions was influential in determining inclusion status of each question. Consideration was given to question relevance across settings, in different levels of health facilities, for different levels of staff (ensuring a mix that was relevant to clinical and non-clinical staff), ease of translation and clarity of understanding. For example, if a question was not understood properly in one country, or it required additional explanation by interviewers, then there was a higher likelihood that the question was removed. In some sites, where questions were deemed important to retain in the brief questionnaire, but where choice of wording had compromised comprehension in some sites, the group rephrased the question based on recommendations from the field teams.

## Results

The main result of this collaborative process was a brief questionnaire that measures actionable drivers of stigma within health facilities.

### Questionnaires

The outcome of the content-development workshop was the field-tested questionnaire that combined the groups’ prioritized questions in each domain, plus background demographic information (see Table 1 for details of specific items). This questionnaire [28] has 18 core questions and, with sub-items included, 71–95 total items, depending on skip patterns (inclusive of the module). Workshop participants were also asked to identify any critical gaps in existing measures. Stigma towards key populations and health facility policies were two identified gaps. Questions were developed and field-tested to fill these gaps. In addition, workshop participants developed a module for measuring stigma towards pregnant women living with HIV to be implemented only among health facility staff providing services to pregnant women because of the added potential negative consequences of stigma for the health of pregnant women living with HIV and vertical transmission of HIV [33].

The finalized brief questionnaire for research and evaluation [34] is summarized in Table 3, which shows how many, and in which sections, questions were reduced from the field-tested questionnaire. This questionnaire has 17 core questions and, with sub-items included, 39–49 total items, depending on skip patterns (inclusive of the module). The questionnaires are available in five languages – Arabic, Chinese, English, Spanish and Swahili – along with an implementation guide in English. These are available at www.healthpolicyproject.com.

**Table 3:** Results of questionnaire item reduction by question types and totals

Section	Category	Field-tested questionnaire	Final brief questionnaire
Background section	Demographic	6 Questions	2 Questions
	Job duties and facility-related	9 Questions; 1 with 9 sub-items	5 Questions; 1 with 4 sub-items

Drivers	Health facility policies and work environment	7 Questions; 1 with 6 sub-items	5 Questions; 1 with 2 sub-items
	Fear	1 Question with 9 sub-items	1 Question with 4 sub-items^1^
	Attitudes towards PLHIV	1 Question with 6 sub-items	1 Question with 5 sub-items; 1 Question about HIV-positive women's right to have babies
	Shame	2 Questions	0 (as included as a sub-item in attitude question)
	Willingness to treat key populations	1 Question with 6 sub-items, each sub-item had, depending on answer, 4 additional possible questions	3 Questions focused on key populations of MSM, Sex workers and PWID. Each question has three possible sub-items, depending on answer

Enacted stigma	Observed	1 Question with 8 sub-items	1 Question with 3 sub-items
	Extra infection precautions	1 Questions with 6 sub-items	1 Question with 4 sub-items
	Secondary stigma	1 Question with 4 sub-items	1 Question with 3 sub-items^1^

Module: stigma towards pregnant women living with HIV among facility staff who care for pregnant women	Fear	1 Question with 2 sub-items	1 Question
	1 Question with 7 sub-items	1 Question with 4 sub-items	
	Observed	1 Question with 5 sub-items	1 Question with 5 sub-items

1 These questions are asked differently in high-prevalence and low-prevalence settings.

### Field questionnaire data

Data for the combined sample across the six sites (*n =* 1893) include the percentages for the country mean and ranges. (Each site will report separately on their individual results in future publications.) For several items large ranges were observed, a reflection of the diversity across the sites which includes HIV prevalence and health systems. The mean age of all respondents was 37.5 years, ranging from 32.5 to 40 years. The majority of respondents were female (mean=71.8%) ranging from 56.3 to 82.1%.

Table 4 presents the percentage mean and ranges for selected questions capturing drivers of stigma that were included in the brief questionnaire. Roughly, one in four respondents disagreed with the statement “I would never test a patient for HIV without the patient's informed consent.” More than half of respondents (54.5%) reported policies to protect PLHIV from discrimination in a facility. In terms of worry of HIV acquisition when caring for or providing services to PLHIV, as invasiveness of the procedure increased, worry also increased. On items in the attitude scale, the mean percentage agreement varied from a low of 15.7% for the statement “People living with HIV should feel ashamed of themselves” to 40.6% agreement to the statement “most people living with HIV do not care if they infect other people.”

**Table 4:** Stigma drivers, percentages and country ranges (*n=*1893)^1^

Health facility policies and work environment				

Level of agreement with the following statements^2^		Agree	Disagree	Do not know
I would never test a patient for HIV without the patient's informed consent	Mean	72.4	23.2	0.5
	Range	38.7–92.0	5.3–58.3	0.0–3.0
There are adequate supplies (e.g., gloves) in my health facility that reduce my risk of becoming infected with HIV	Mean	80.7	16.8	0.7
Range	53.7–96.7	2.3–46.3	0.0–4.0
There are standardized procedures/protocols in my health facility that reduce my risk of becoming infected with HIV	Mean	73.0	24.3	0.2
Range	10.0–93.4	5.3–88.7	0.0–1.3
		Yes	No	Do not know
My health facility has policies to protect patients living with HIV from discrimination	Mean	54.5	24.1	21.2
	Range	1.7–84.1	4.3–97.7	0.7–47.2
How hesitant are healthcare workers in this facility to work alongside a co-worker living with HIV regardless of their duties?^3^		Hesitant	Not hesitant	Do not know
Mean	51.5	42.3	0.4
	Range	22.0–83.4	16.3–75.3	0.0–2.7

**Worry related to contracting HIV when caring or providing services to people living with HIV**				

**Level of worry when conducting the following activities** 4		**Worried**	**Not worried**	

Took the temperature of a patient living with HIV (*n=*1205)	Mean	15.3	82.4	
	Range	5.3–43.4	56.6–90.5	
Touched the clothing of a patient living with HIV (*n=*1672)	Mean	23.3	74.7	
	Range	6.2–57.2	42.8–91.5	
Dressed the wounds of a patient living with HIV (*n=*1061)	Mean	59.6	37.5	
	Range	38.8–85.7	14.3–51.0	
Drew blood from a patient living with HIV (*n=*1052)	Mean	67.0	42.5	
	Range	44.1–83.0	17.0–49.6	

**Opinions about people living with HIV**				

**Level of agreement with the following statements** 2		**Agree**	**Disagree**	**Do not know**

HIV is a punishment for bad behaviour	Mean	16.3	82.1	
	Range	3.9–54.3	45.7–91.0	
Most people living with HIV do not care if they infect others	Mean	40.6	57.3	
	Range	15.0–69.0	31.0–84.4	
People living with HIV should feel ashamed of themselves	Mean	15.7	82.8	
	Range	5.3–54.7	45.3–94.7	
Most people living with HIV have had many sexual partners	Mean	35.8	62.4	
	Range	17.7–68.0	32.0–81.7	
People get infected with HIV because they engage in irresponsible behaviours	Mean	38.1	59.8	
	Range	21.1–69.0	31.0–78.0	
People living with HIV should be allowed to have babies if they wish	Mean	56.7	39.6	0.3
	Range	13.3–90.3	9.4–84.7	0.0–0.2
If I had a choice, I would prefer not to provide services to people who inject illegal drugs (*n=*1593)	Mean	17.6	78.4	
Range	11.9–35.7	64.3–85.1	
If I had a choice, I would prefer not to provide services to men who have sex with men (*n=*1593)	Mean	13.1	83.0	
Range	3.0–27.0	73.0–95.0	
If I had a choice, I would prefer not to provide services to sex workers (*n=*1593)	Mean	12.4	83.8	
	Range	5.7–29.7	70.3–93.4	

1 (*n*–1893) applies to each category, unless otherwise noted; % may not add to 100 because of missing data.

2 Response categories: strongly agree, agree, disagree, and strongly disagree; results collapse responses.

3 Response categories: very hesitant, somewhat hesitant, a little hesitant, and not hesitant; results collapse responses.

4 Response categories: very worried, worried, a little worried, a not worried; results collapse responses.

Table 5 presents the percentages for the mean and ranges of questions measuring enacted stigma that were included in the final questionnaire. The mean percent of respondents who reported observing a *healthcare worker talking badly about PLHIV or thought to be a PLHIV* was 29.9%. Use of extra infection precautions is present with 30.9% reporting wearing double gloves. Secondary stigma, however, is relatively low probably due to the fact that five of the six sites are in low HIV prevalence settings.

**Table 5:** Enacted stigma, combined percent (*n=*1893) and country ranges

Observed stigma (*n=*1853)			

In<12 months how often observed the following at your health facility^1^		At least once (%)	Never (%)
Healthcare workers unwilling to care for a patient living with HIV	Mean	23.4	74.4
	Range	12.7–43.1	56.9–87.3
Healthcare workers providing poorer quality of care to a patient living with HIV than to other patients	Mean	20.1	77.5
	Range	8.3–28.5	68.7–91.7
Healthcare workers talking badly about people living with or thought to be living with HIV	Mean	29.9	67.5
	Range	14.0–58.5	41.5–86.0

**Infection precaution measures**			

**Typically use any of the following measures when providing services to a patient living with HIV:**		**Yes (%)**	**No (%)**

Avoid physical contact (*n=*1575)	Mean	26.8	69.6
	Range	6.4–69.4	30.6–87.2
Wear double gloves (*n=*1506)	Mean	30.9	66.1
	Range	19.0–48.2	51.8–79.9
Use any special measures that you do not use with other patients (*n=*1495)	Mean	26.9	69.1
	Range	7.2–50.5	49.6–83.3

**Experiences with secondary stigma (*n=*1814)**			

**In the past 12 months, how often have you^1^:**		**At least once (%)**	**Never (%)**

Experienced people talking badly about you because you care for patients living with HIV	Mean	12.2	81.3
	Range	5.0–34.6	65.1–95.0
Been avoided by friends and family because you care for patients living with HIV	Mean	4.8	88.6
	Range	1.3–9.4	72.6–97.3
Been avoided by colleagues because of your work caring for people living with HIV	Mean	2.6	90.6
	Range	1.0–5.1	73.0–98.2

1 Response categories included most of the time, several times, once or twice and never.

### Attitude towards PLHIV scale

Table 6 presents the factor loadings for the attitude scale and reliability of the scale by country. The alpha for the combined sample was 0.78. Across all six countries only one factor formed but the items in the factor varied. In Kenya, Dominica and St. Christopher & Nevis all six items loaded on to the single factor, whereas in Puerto Rico and China, “PLHIV could have avoided HIV if they wanted to” (Q27a) did not load and in Egypt, “Most PLHIV do not care if they infect other people” (Q27c) did not load on the factor. While both items had reasonable variability across each country, during the content-development workshop, persons living with HIV stressed the importance of Q27c. Furthermore, the analysis workshop participants felt that Q27a was captured in another item “People get infected with HIV because they engage in irresponsible behaviors” (Q27f), and therefore, concluded to drop Q27a and keep Q27c in the attitude scale.

**Table 6:** Attitude scale: factor loadings and reliability

5-item attitude scale	China	Dominica	Egypt	Kenya	Puerto Rico	St. Christopher & Nevis
People living with HIV could have avoided HIV if they had wanted to (Q27a)	–	0.5340	0.6828	0.4588	0.3415	0.5657
HIV is a punishment for bad behaviour (Q27b)	0.5950	0.6155	0.8013	0.5152	0.6770	0.5302
Most people living with HIV do not care if they infect other people (Q27c)	0.3501	0.4383	–	0.4586	0.6202	0.6139
People living with HIV should feel ashamed of themselves (Q27d)	0.7047	0.6072	0.7308	0.4159	0.6513	0.4967
Most people living with HIV have had many sexual partners (Q27e)	0.5627	0.6434	0.6862	0.6463	0.6061	0.6759
People get infected with HIV because they engage in irresponsible behaviours (Q27f)	0.7078	0.6307	0.7737	0.6227	0.5869	0.5977

**Cronbach's *α* 5-item scale of Q27b–Q27f**	0.72	0.73	0.77	0.67	0.76	0.73

## Discussion

The results of this international multi-site collaborative effort demonstrate that it is possible to have a brief, standardized programmatic tool to measure stigma within health facilities that works well across diverse country contexts, prevalence areas, languages, healthcare settings and health worker types. The results (Tables 4 and 5) also demonstrate that while varying across sites, stigma is still prevalent across both the high- and low-prevalence sites and that there is still much work to be done to create a facility environment that fosters the delivery of stigma-free services. For example, the mean across all sites for agreement with the statement “most people living with HIV do not care if they infect others” was 40.6%, while only a little over half (54.5%) of respondents reported that their facilities had policies in place to protect patients living with HIV from discrimination. More than a third of respondents (39.6%) disagreed with the statement “People living with HIV should be allowed to have babies if they wish.” Respondents also report that they have observed healthcare workers unwilling to care for a patient living with HIV in their facility in the past 12 months (23.4% across sites) and a third (30.9%) report that they use double gloves when providing services to patients living with HIV.

The content of this tool is grounded in previous work measuring stigma among health providers, both on the level of individual questions and around the larger thematic areas of the questionnaire. Field testing of this instrument confirmed that the key domains measured and a sub-set (or similar) of the individual questions tested in previous work in single sites [8, 9, 11, 13, 14, 16, 17] worked across diverse contexts. To the best of our knowledge, only one other study [35] has tested measurement among a group of health providers (nurses) across multiple country sites (Lesotho, Malawi, South Africa, Swaziland and Tanzania). While all sites were in East and Southern Africa [13, 36], this work also demonstrated that use of a standard stigma data-collection tool for health providers across differing contexts is feasible. While not specific to healthcare providers, the work of Genberg et al. [37] also illustrated that a standard measurement tool for stigma can work across diverse settings (Thailand, Tanzania, South Africa and Zimbabwe) in the general population.

While the process demonstrated that a core set of questions works well to measure key domains for stigma-reduction programming in health facilities across diverse settings, the implementation process yielded several lessons, including lessons about the content of specific questions. This led to certain questions being dropped from the brief questionnaire, or if deemed too important to drop for programmatic reasons, being rephrased based on the field-testing experience. For example, asking about fear of HIV transmission in a high-prevalence context where many of the respondents may be living with HIV was problematic as phrased in the piloted questionnaire. Conversely, asking respondents about experiences of secondary stigma in low-prevalence settings had little relevance because respondents in these contexts provided care to so few PLHIV that it was unlikely anyone else would know to stigmatize them. However, while actual experience of secondary stigma was not particularly relevant in low-prevalence contexts, the anticipation that this might happen was considered relevant. These two issues were resolved by offering different question wordings for low- or high-prevalence HIV settings.

In addition, a few of the factor loadings and the Cronbach’s *α* for the opinion scale were slightly lower for Kenya than the other sites. As Kenya was the only high HIV-prevalence field-testing site, it could be that this reflects the respondents’ longer experience and exposure to HIV and HIV programming, higher likelihood of personally knowing PLHIV, or possibly the fact that a sub-set of the respondents were likely living with HIV. Implications for framing of attitudinal questions (apart from the distinctions described above) are unclear, however, in the absence of more field testing in additional high-prevalence countries.

The questions that were deemed too important to drop, but needed re-wording based on the field implementation experience, came from two domains that were identified as gaps during the initial content-development meeting – key populations and facility policies. They therefore comprised new questions developed by the meeting participants, as opposed to questions that had already been tested in other instruments.

An example of a facility policy question that did not work well as phrased was: “My health facility has policies to protect patients living with HIV from discrimination (response categories: Yes, No, DK).” The challenge with this question was a lack of specificity in the understanding or interpretation of what a *policy* means across the sites. The question was thus rephrased to read: “My health facility has written guidelines to protect patients living with HIV from discrimination.” Another question that required re-wording focused on willingness to provide services to a specific key population. The piloted version of the question had the following question stem: “Please tell us it you strongly agree, agree, disagree, or strongly disagree with the following statement in relation to each group listed in the table below. I would prefer not to provide services to …” (and then listed multiple key population groups). The challenge discovered with this question was that despite the use of the word “prefer,” respondents answered that they would provide services (even if they preferred not to) because they did not think they had a choice in the matter. Based on recommendations from the field testing experience, the question was re-worded to read: “If I had a choice, I would prefer not to provide services to ….”

On the implementation side, key lessons learned focused on mode of administration (self- or interviewer-administered). For example, in Egypt all data were collected through interviewer-administered questionnaires, as that was deemed most context-appropriate, while in other sites a mixture of self- and interviewer-administered was most appropriate. Anonymity was also of concern in some sites even though no identifiers were collected and self-filled questionnaires were returned in manner that ensured confidentiality. This concern seems to have stemmed from the set of background questions asked and worry that somehow this information could be pieced together to identify a particular respondent. This was of particular concern in the two island nations where small populations meant that almost half of all staff working in the health facilities in the country were interviewed. To respond to this concern, the brief questionnaire now includes only a limited number of essential background questions and the recommendation that implementers use a facility code if they require specific information on types of facilities, rather than asking respondents for this information. In Puerto Rico, half the self-administered sample was delivered with paper and pencil, the other using iPads. While further analysis needs to be conducted, the initial feedback indicates that use of iPads provides a better method of administration, both peaking respondents interest in participating in order to use the technology while also providing more trust in the anonymity of the questionnaire. In addition, the automatic skip patterns in the iPad questionnaire ensured ease of completion and reduced errors.

### Limitations

The process did have limitations. The purpose of this effort was to demonstrate feasibility and applicability of a shortened tool that could be used in programmatic applications across a diverse set of contexts and languages. It had to allow for variability by site in some key factors, and be responsive to resource constraints. It therefore was not conducted in accordance with standard methodology for scale validation. For example, the health worker sampling methods varied across sites, sites varied in their mode of administration, and the tool was not validated against any similar constructs or outcomes. As with any data collection on sensitive issues, there can be social desirability bias in responses, and this appears to have manifested in non-response to several questions in the Caribbean sites, where the most concerns around confidentiality emerged due to small size of the health facility workforce. Interestingly, the questions that field staff indicated as most likely to be subject to social desirability bias were questions that respondents perceived would put the facility, rather than themselves, in poor light. For example, some participants responded that gloves were always available in the facilities, when the research team in fact knew they were not. While the questionnaire was field tested in six sites covering diverse contexts and in multiple languages, these sites are not fully representative of all regions or languages of the world, and five of the six sites were low HIV-prevalence contexts. Therefore, it may be important to conduct brief pilots when implementing the tool in new contexts or languages to determine the interpretability of the new translation and appropriate mode of administration.

While there are some limitations with the tool, it also has many strengths including: covering the key HIV stigma domains shown to be important for stigma-reduction programming in health facilities in a brief manner; being evidence-based, drawing on validated tools from the literature; and successful administration in multiple diverse country settings and languages. A particular strength is the shorter length of the questionnaire, which is important for busy and resource-constrained health facilities. The reduced length also allows the questionnaire to be used as a stand-alone tool in routine monitoring, and/or as part of a larger evaluation of country-level or health facility-level activities.

## Conclusions

The purpose of this study was to develop and test a standardized tool that assesses HIV stigma in healthcare settings. The development, field testing and analysis process carried out by this team demonstrate that a brief yet comprehensive instrument that captures key domains of stigma for programmatic action can be successfully implemented across diverse settings and provide consistent and robust results. The brief tool is now available for government officials, policy makers and programmers to determine the amount of HIV stigma in health facilities, design evidence-based programming responses to reduce stigma, monitor stigma over time, and evaluate the effects of stigma-reduction interventions and programmes. There is potential for wide use of this tool, both as a stand-alone survey or integrated within other health facility surveys. Areas of future work for this tool are to observe how it performs with repeated administrations over time, in additional contexts (particularly high-prevalence settings), and to triangulate data collected in health facilities on stigma and discrimination with data being collected among PLHIV and key population clients of health facilities, for example by the stigma index programme (http://www.stigmaindex.org/). Further work is needed to test and expand questions measuring stigma towards key populations.

Institutionalizing the measurement of stigma as routine practice, and doing so on a large scale, could strengthen the delivery of high-quality care, improve patient outcomes and satisfaction, improve the work environment for health facility staff, and increase the effectiveness of investments in HIV prevention, care and treatment. This brief tool can thus contribute to addressing HIV stigma within health facilities and towards progress in ensuring that PLHIV, and people often associated with HIV, receive high-quality health services and that their rights and privacy are upheld.
